# Patient with Swollen Neck

**DOI:** 10.5811/cpcem.2019.1.41294

**Published:** 2019-01-29

**Authors:** Akshay K. Elagandhala, Robert Liu, Henry E. Wang

**Affiliations:** University of Texas Health Science Center at Houston, Department of Emergency Medicine, Houston, Texas

## CASE PRESENTATION

**Image 1:**
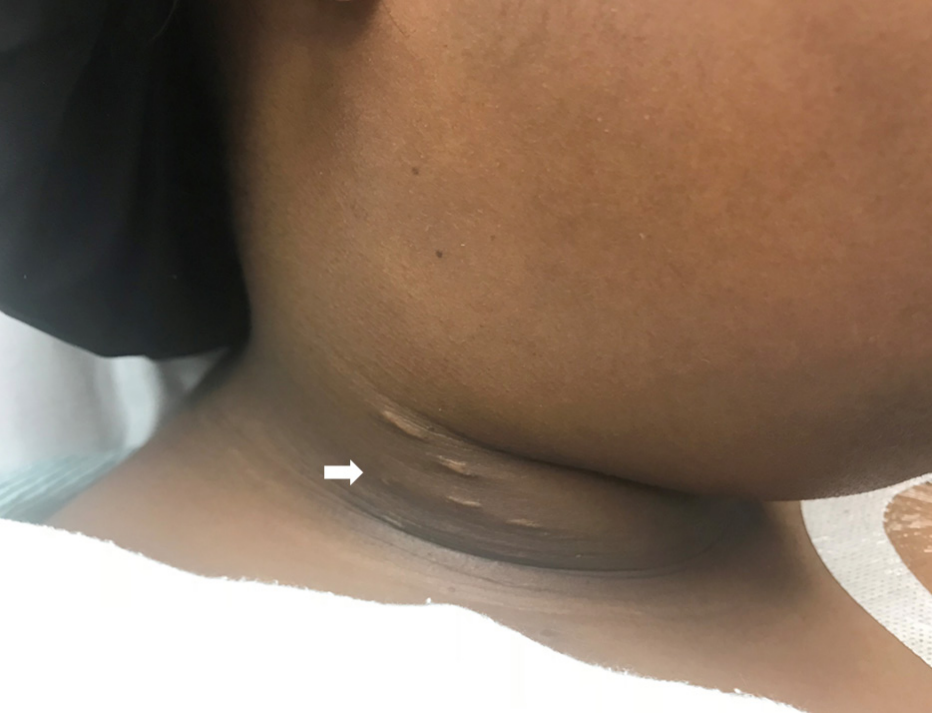
External view of the right neck swelling. Arrow indicates area of swelling.

**Image 2:**
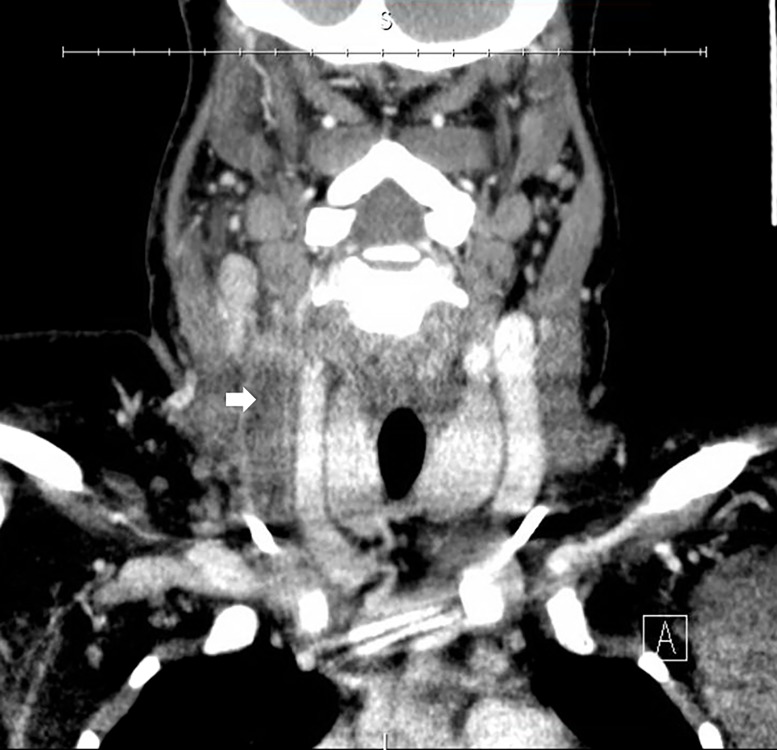
Coronal view of a computed tomography of the neck with contrast. Arrow indicates thrombus in the right internal jugular vein with complete occlusion.

A 25-year-old female with a history of sickle cell disease (on prophylactic penicillin VK) and venous thromboembolic disease (on oral anticoagulation with apixaban) presented to the emergency department with one week of right-sided neck pain and subjective fevers, and a one day history of trismus. Physical examination revealed warmth, swelling and tenderness to the right lateral neck near the angle of the mandible, with associated mild trismus (Image 1). Additional history revealed the presence of bilateral, subclavian, implanted venous access ports. We ordered a computed tomography (CT) of the neck with intravenous (IV) contrast (Image 2).

## DISCUSSION

The CT revealed complete occlusion of the right internal jugular vein consistent with Lemierre’s syndrome.^1^,^2^ Lemierre’s syndrome is acute thrombophlebitis of the internal jugular vein, usually associated with direct spread of adjacent odontogenic or oropharyngeal bacterial infections. The most common microbial source is the gram-negative, anaerobic *fusobacterium* species, a part of the oropharyngeal flora. Conditions that should be considered in the work-up of neck swelling may include retropharyngeal abscess, Ludwig’s angina, subcutaneous abscess, suppurative parotitis, odontogenic infection (e.g., periodontal abscess, submandibular osteomyelitis), peritonsillar abscess, pharyngitis with reactive cervical lymphadenopathy, malignancy (e.g., lymphoma), and carotid/vertebral artery dissection.

Management of Lemierre’s syndrome includes antibiotic therapy and selective use of anticoagulation. Initial antibiotic coverage should include a beta-lactamase resistant antibiotic with anaerobic coverage (e.g., ampicillin and sulbactam, or piperacillin and tazobactam) to cover for oropharyngeal flora. Coverage for methicillin-resistant gram-positive species should also be considered (e.g., vancomycin and fluoroquinolones), especially in catheter-associated infections.^3^,^4^ In select cases, surgical abscess drainage or jugular vein ligation may be necessary.

In this case, blood cultures drawn from both ports indicated the presence of methicillin-resistant *staphylococcus epidermidis*, presumptively from colonization of the venous access ports. No additional microbial source was identified. The patient’s history of sickle cell disease, venous thromboembolic disease, and the presence of venous access ports likely potentiated the risk of internal jugular vein thrombus formation despite concurrent long-term anticoagulation. The patient was hospitalized, received antibiotic therapy with vancomycin and cefepime (later changed to ampicillin-sulbactam) and anticoagulation with IV heparin, and underwent surgical removal of both internal jugular venous access ports.

**CPC-EM Capsule**

What do we already know about this clinical entity?

*Odontogenic and oropharyngeal infections may cause thrombophlebitis of the jugular vein (Lemierre’s syndrome)*.

What is the major impact of the image(s)?

*This image depicts complete internal jugular vein occlusion consistent with Lemierre’s syndrome*.

How might this improve emergency medicine practice?

*Clinicians should consider Lemierre’s syndrome in individuals presenting with neck pain and fever. The risk may be heightened in those with a history of hematologic or venothromboembolic disease or an indwelling vascular catheter*.
